# Blood Lead Levels and Associated Factors among Children in Guiyu of China: A Population-Based Study

**DOI:** 10.1371/journal.pone.0105470

**Published:** 2014-08-19

**Authors:** Pi Guo, Xijin Xu, Binliang Huang, Di Sun, Jian Zhang, Xiaojuan Chen, Qin Zhang, Xia Huo, Yuantao Hao

**Affiliations:** 1 Department of Medical Statistics and Epidemiology, School of Public Health, Sun Yat-sen University, Guangzhou, China; 2 Laboratory of Environmental Medicine and Developmental Toxicology, and Guangdong Provincial Key Laboratory of Infectious Diseases and Molecular Immunopathology, Shantou University Medical College, Shantou, China; 3 Good Clinical Practice Office, Cancer Hospital of Shantou University Medical College, Shantou, China; Gentofte University Hospital, Denmark

## Abstract

**Objectives:**

Children's health problems caused by the electronic waste (e-waste) lead exposure in China remains. To assess children's blood lead levels (BLLs) in Guiyu of China and investigate risk factors of children's elevated BLLs in Guiyu.

**Material and Methods:**

842 children under 11 years of age from Guiyu and Haojiang were enrolled in this population-based study during 2011–2013. Participants completed a lifestyle and residential environment questionnaire and their physical growth indices were measured, and blood samples taken. Blood samples were tested to assess BLLs. Children's BLLs between the two groups were compared and factors associated with elevated BLLs among Guiyu children were analyzed by group Lasso logistic regression model.

**Results:**

Children living in Guiyu had significant higher BLLs (7.06 µg/dL) than the quantity (5.89 µg/dL) of Haojiang children (*P*<0.05). Subgroup analyses of BLLs exceeding 10 µg/dL showed the proportion (24.80%) of high-level BLLs for Guiyu children was greater than that (12.84%) in Haojiang (*P*<0.05). Boys had greater BLLs than girls, irrespectively of areas (*P*<0.05). The number of e-waste piles or recycling workshops around the house (odds ratio, 2.28; 95% confidence interval [CI], 1.37 to 3.87) significantly contributed to the elevated BLLs of children in Guiyu, and girls had less risk (odds ratio, 0.51; 95% CI, 0.31 to 0.83) of e-waste lead exposure than boys.

**Conclusions:**

This analysis reinforces the importance of shifting e-waste recycling piles or workshops to non-populated areas as part of a comprehensive response to e-waste lead exposure control in Guiyu. To correct the problem of lead poisoning in children in Guiyu should be a long-term mission.

## Introduction

Discarded electrical and electronic equipment and components, known as electronic waste (e-waste), have brought an emerging impact on global environment due to that they are the most rapidly increasing sources of waste worldwide [1]. Accordingly, a large proportion of e-waste was transferred to less developed countries for dumping or recycling [2]. The globalization of e-waste has certainly adverse environmental and health impacts, bringing an elevated exposure to hazardous substances to workers who involving daily e-waste recycling activities. The activities of e-waste recycling made the involved population exposed to various chemical contaminations, putting them at risk of taking in or ingesting of contaminated water, air, and food supplies in their living areas [3]. It is well characterized the children aged below 7 years are particularly vulnerable to lead poisoning mainly due to their immature central nervous systems [4]–[6]. Although the adverse effects of exposure to lead have been understood, significant concerns remain about risk factors associated with elevated BLLs in children living in China.

**Figure 1:**
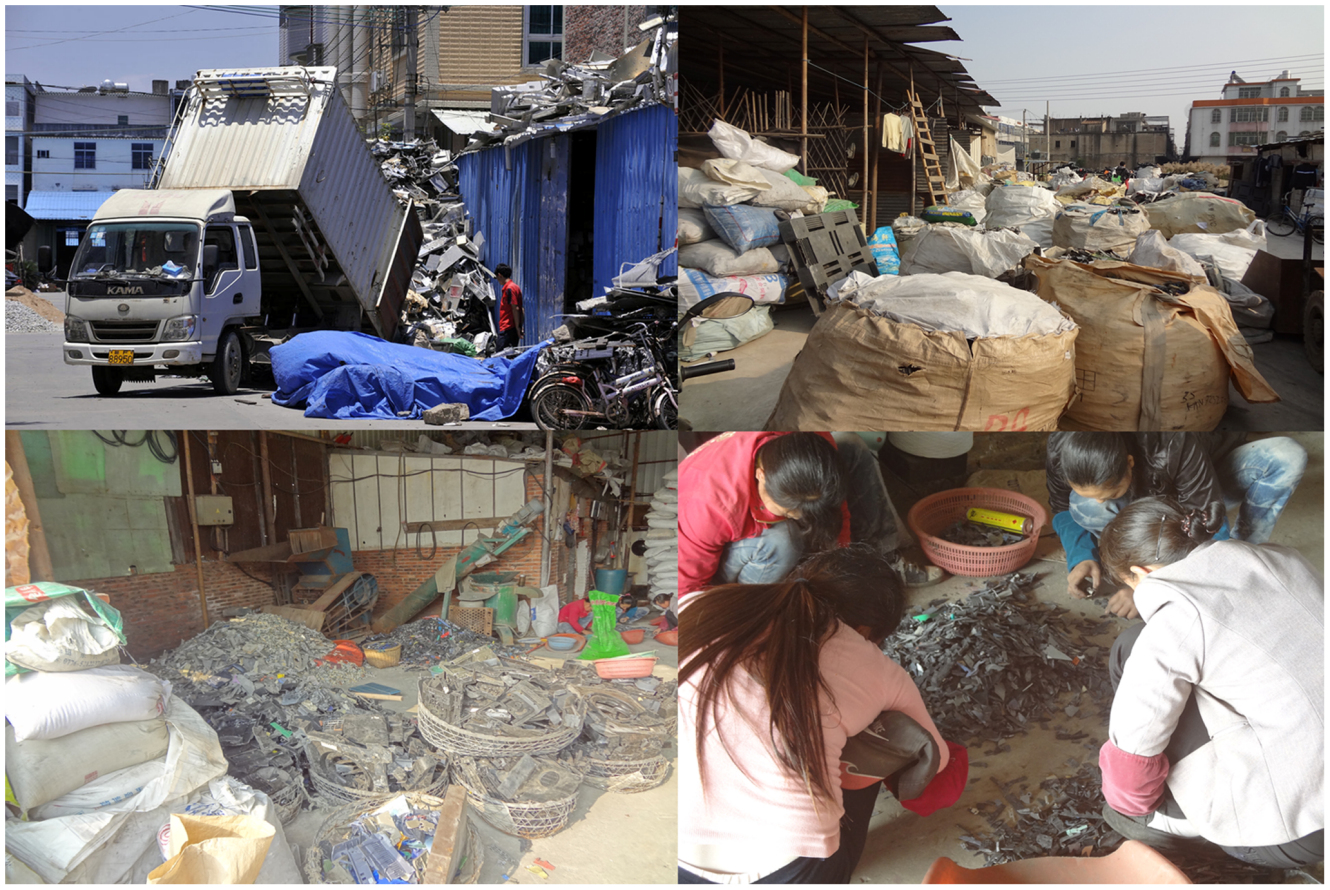
The current status of e-waste processing in Guiyu.

As a famous e-waste recycling site, Guiyu is located in Shantou City, in the southeastern coast of Guangdong province in China. There are 28 villages with a total area of 52 km^2^ and a resident population of 132,000 in Guiyu. It was reported to have nearly 60–80% of families engaging in e-waste recycling [7]. In Guiyu, processing e-waste to extract valuable metals for sale or reuse is a dominant industry for the local residents. E-waste processing in Guiyu has caused serious health problems to the natives, including adverse birth outcomes [8], [9], decreased pulmonary function [10], and child temperament alterations [11]. According to our survey (Figure 1), piles of e-waste are continuously transported to the destination for recycling in Guiyu, and most e-waste recycling currently occurs in small scale family-based workshops where the workers are engaged in unsafe recycling practices without the benefit of exposure-minimizing technology or protective equipment. The recycling procedures of e-waste are relatively primitive. Kids are frequently seen involving the activities of e-waste processing or playing with the discarded electronics in the sites. The exposure to lead caused by e-waste recycling activities is an urgent public health problem for the natives, which makes them have significant high blood lead levels (BLLs) even at poisoning one [7]. National surveillance data reveal that the average BLLs in urban children aged 0 to 6 in China decreased from 7–10 µg/dL to 2.5–6 µg/dL from the end of the 1990s through 2009 [12], [13]. Based on our previous reports, the BLLs of children in Guiyu had decreased from 15.3 µg/dL to 7.30 µg/dL over the past five years [7], [11], [14], [15]. The e-waste lead exposure of children in Guiyu has declined substantially, but the figure is still higher than the national average. Guiyu is still facing the serious e-waste lead exposure problem in China.

However, there has been a potential change of lead-polluting industries in Guiyu and an increased concern about the effects of lead exposure on children's health in recent years. Understanding risk factors that contribute to the elevated BLLs of local children can help us to take intervention measures. We carried out a population-based study to re-evaluate the BLLs for local children. This work aims to provide an up-to-date assessment of children's BLLs, and identify risk factors associated with the elevated BLLs among children.

## Material and Methods

### Ethics Statement

This study was approved by the Human Ethics Committee of Shantou University Medical College.

### Study population

A total of 842 children under 11 years of age who met the inclusion criteria from two native kindergartens in Guiyu and Haojiang were enrolled. Haojiang was selected as a reference area of non e-waste processing activities due to its density of population and traffic, residential lifestyle, cultural background and socioeconomic status were comparable to those of Guiyu. The Dutou kindergarten from Guiyu and Haijun kindergarten from Haojiang are the local biggest kindergartens, respectively. Children from the both two kindergartens are more widespread and the samples are representative of children living in the study areas. For another, to monitor the BLLs of local children for a long term and track the dynamic changes, we chose to recruit children from the kindergartens. Children attended the survey were randomly selected in each kindergarten under the permission of parental consent to ensure the absence of selection bias. The first study phase occurred from December 2011 to March 2012, when 342 children were enrolled from Guiyu (n = 183) and Haojiang (n = 159). The second phase occurred between December 2012 and January 2013, when 330 and 170 children were enrolled from the same kindergartens in the two areas, respectively. The duplication of data from these two recruitments was checked and removed based on the recorded information including children's name and classroom to ensure the independence of each observation in the Logistic regression model. Children's parents were informed the details of this study, and gave their written informed consents after receiving detailed explanations of the study and potential consequences prior to enrollment. After receiving informed consents, we collected the venous blood and measured the physical growth indices from children. Children's parents were requested to fill in a structured questionnaire.

### Blood sample collection and assay

A total of 6 ml venous blood from each child was collected by trained nurses. Whole blood was stored in vacutainer with EDTA-K2 as anticoagulant. The blood was stored at a −20°C refrigerator and used to determine BLLs by graphite furnace atomic absorption spectrometry (GFAAS, Jena Zeenit 650, Germany). All plastic tubes used for blood lead tests were washed thoroughly, soaked with dilute nitric acid, rinsed with deionized water and dried before using.

### Questionnaire survey

A lifestyle and residential environment questionnaire was used to investigate the potentially associated factors. It mainly included children's age, sex and physical growth indices, and 25 questions involving children's behavior habits (frequency of outdoor playing, washing hands before eating, hand-to-mouth activity), dieting habits (frequency of eating preserved eggs, dairy and bean products, canned foods), their parents' smoking habits and socioeconomic status (annual household income, job, and education level), housing environment (distance that the house stands from the nearest road, and whether e-waste recycling workshops stand around the house), residential history and source of drinking water. Children's growth indices (height, weight, head and chest circumferences) were immediately measured on the sites. The questions for the questionnaire were selected based on our previous studies [7], [8] about the risk factors for lead exposure in the Chinese pediatric population.

To identify risk factors of elevated BLLs, this analysis ended up with 334 questionnaires after checking the validity of each questionnaire. Each question was coded as a numeric score variable. The description of the surveyed factors was listed (Table S1 in File S1.).

### Statistical analyses

#### Comparison of children's BLLs

Initially, data that normally distributed were expressed as mean and standard deviation, and data with skewed distribution were expressed as median and interquartile range. For comparative analysis, the log-transformed values of BLLs were calculated and used. Participant characteristics and main investigated factors (children's behavior habits, dietary habits, living conditions, household income, parents' educational level and city of residence) of children with high (≥10 µg/dL) and low (<10 µg/dL) BLLs were compared. Independent-sample *t*-tests were used for testing the differences of numeric variables between two groups of children, and Chi-square tests for categorical data. Covariance analysis was performed to test for significance of the differences in BLLs between Guiyu and Haojiang children. The BLLs of boys and girls were compared using the Mann-Whitney U method. SPSS software 13.0 (SPSS, Inc., Chicago, IL, USA) was used for the statistical analyses. A *P*-value <0.05 was considered to be statistically significant.

#### Identification of BLLs relevant factors

According to the U.S. Centers for Disease Control and Prevention, BLLs exceeding 10 µg/dL means public health actions should be prompted [16]. The data of BLLs from Guiyu were divided into two groups: high-level group (BLLs≥10 µg/dL) and low-level group (BLLs<10 µg/dL). We performed a variable selection model to identify the factors that can significantly distinguish subjects with high BLLs from that with low ones.

Due to the categorical characteristics, each covariate involving three or more categories was initially converted to two or more dummy variables. For example, the covariate ‘Whether the child often washes hands before eating meals or snacks’, had 4 categories in the questionnaire: no, occasional, often and always. Hence 3 dummy variables were introduced in the model representing the last three categories referenced on the first category. After coding, there were 72 covariates used for further analysis. A Lasso-based penalization estimation method can be applied to study risk factors of diseases, because the Lasso is an efficient algorithm of variable selection [17], [18]. Suppose that in a logistic regression set-up we have an *n*×1 binary response vector *Y*, an *n*×*p* design matrix *X* and a *p*-dimensional parameter vector 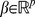. The Lasso estimator is defined as (1): 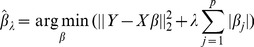
(1)where 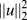 denotes 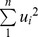
for a vector 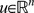, and 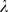 is the tuning parameter, which can shrink some components of 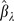
to zero when the value of 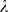 is large.

However, it is reasonable to select potential categorical covariates at the group level. Therefore the group-wise Lasso variable selection method should be used. In this study we proposed to perform a group Lasso logistic regression model [19] to identify risk factors of e-waste lead exposure. As an extension of the Lasso, the group Lasso method selects whole factors instead of individual dummy variables by modifying the penalty function [19]. Assume that in a group Lasso logistic model, the observations 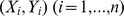 denote a *p*-dimensional vector 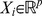
of *G* predictors and a binary response variable 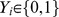. The degree of freedom of the *g*th predictor is denoted by 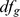, and 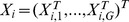
represents the group of variables. To model the conditional probability 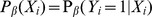
_,_ the formula (2) was used: 
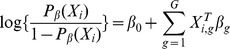
(2)where 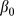 is the intercept and 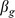 is the parameter vector corresponding to the *g*th predictor. The estimator 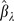 can be given by minimizing the convex function (3): 
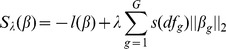
(3)where 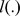 is the log-likelihood function of 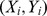 in the model. The 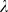 is the tuning parameter, which controls the amount of penalization in the group Lasso model. The rescaling function 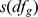 is used to rescale the penalty, and the 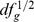 is proposed to estimate the function [19].

In this work, the parameter estimations in the group Lasso were computed via a group descent algorithm [20], which was an efficient approach for fitting models with grouped penalties. We performed 10-fold cross validation over a grid of values for 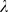, and selected the 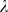 corresponding to the minimum cross-validation deviance. The selected 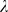 was applied to determine a group of significant covariates. The group Lasso logistic regression model was established using the grpreg package within R software [21] with version 2.13.0.

#### Model validation

In order to confirm whether the selected covariates by the group Lasso method were robust for this study, 100 sets of bootstrapped data were generated from the real survey data, and the group Lasso logistic regression model was performed on the 100 sets of bootstrapped data to do variable selection. The bootstrapped data with various sample sizes (*N* = 150, 200 and 250) were generated to test the consistency of the method. We also investigated whether the association between the selected covariates and the response variable presented in the permutated data. 100 sets of independently permutated data were generated from real data by fixing the proportion of high-level and low-level BLLs subjects during resampling. Recursively, the group Lasso model was employed to do variable selection in the permuted data sets with various sample sizes (*N* = 150, 200 and 250).

## Results

### Main characteristics between children with different BLLs

For the whole sample, the differences in characteristics and major investigated factors between children with high (≥10 µg/dL) and low (<10 µg/dL) BLLs included children's sex, the habit of eating dairy products, e-waste piles around the house and city of residence (Table 1). Boys tended to have high BLLs than girls (*P*<0.05). Children with low BLLs tended to frequently have dairy products than those with high BLLs (27.6%>17.2%, *P*<0.05). The elevated BLLs of children were significantly associated with e-waste piles around the house (*P*<0.05). Children living in Guiyu had a significant greater proportion of elevated BLLs than that in Haojiang (*P*<0.05).

**Table 1:** Comparison of participant characteristics and main factors investigated of children with high (≥10 µg/dL) and low (<10 µg/dL) blood lead levels.

	BLLs<10 µg/dL (N = 658)	BLLs≥10 µg/dL (N = 165)	*P*-value
Age (years, mean±sd)	4.62±1.13	4.73±1.18	0.266
Age group			0.973
* −3*	33 (5.0%)	9 (5.5%)	
* −6*	394 (59.9%)	98 (59.4%)	
* ≥6*	231 (35.1%)	58 (35.2)	
Sex			0.003
* boys*	351 (53.3%)	109 (66.1%)	
* girls*	307 (46.7%)	56 (33.9%)	
Children's habits			
Outdoor playing (hours per day)			0.608
* 0–1*	62 (10.2%)	16 (10.1%)	
* 1–3*	186 (30.5%)	55 (34.6%)	
* ≥3*	361 (59.3%)	88 (55.3%)	
Washing hand before eating			0.963
* no*	36 (5.6%)	10 (6.1%)	
* occasionally*	308 (48.3%)	81 (49.7%)	
* often*	225 (35.3%)	54 (33.1%)	
* always*	69 (10.8%)	18 (11.0%)	
Hand-to-mouth activity			0.839
* no*	345 (54.2%)	85 (52.1%)	
* occasionally*	226 (35.5%)	61 (37.4%)	
* often*	58 (9.1%)	14 (8.6%)	
* always*	7 (1.1%)	3 (1.8%)	
Dietary habits			
Eating preserved eggs			0.102
* no*	491 (77.9%)	124 (76.1%)	
* occasionally*	115 (18.3%)	28 (17.2%)	
* often*	19 (3.0%)	11 (6.7%)	
* always*	5 (0.8%)	0 (0.0%)	
Eating dairy products			0.029
* no*	37 (5.9%)	8 (4.9%)	
* occasionally*	175 (27.9%)	59 (36.2%)	
* often*	242 (38.6%)	68 (41.7%)	
* always*	173 (27.6%)	28 (17.2%)	
Eating canned foods			0.820
* no*	435 (68.6%)	106 (65.4%)	
* occasionally*	185 (29.2%)	52 (32.1%)	
* often*	13 (2.1%)	4 (2.5%)	
* always*	1 (0.2%)	0 (0.0%)	
E-waste piles around the house			0.000
* no*	409 (64.9%)	70 (43.2%)	
* yes*	221 (35.1%)	92 (56.8%)	
Monthly household income (yuan)			0.987
* <1000*	18 (2.9%)	5 (3.1%)	
* 1000–1500*	49 (8.0%)	14 (8.8%)	
* 1500–2000*	84 (13.7%)	21 (13.1%)	
* >2000*	463 (75.4%)	120 (75.0%)	
Mother's educational levels			0.089
* illiteracy*	2 (0.3%)	1 (0.6%)	
* primary school*	77 (12.1%)	30 (18.6%)	
* middle school*	254 (40.0%)	67 (41.6%)	
* vocational schools*	119 (18.7%)	28 (17.4%)	
* high school*	70 (11.0%)	19 (11.8%)	
* undergraduate*	113 (17.8%)	16 (9.9%)	
City of residence			0.000
* Guiyu*	373 (56.7%)	123 (74.5%)	
* Haojiang*	285 (43.3%)	42 (25.5%)	

### Children's BLLs between Guiyu and Haojiang

Table 2 shows the demographic characteristics and children's BLLs. Children's age, height and weight between Guiyu and Haojiang were different (all *P*<0.05), but no significant differences in the characteristics of sex, head and chest circumference were found between Guiyu and Haojiang children (all *P*>0.05). The median BLLs of Guiyu children was 7.06 µg/dL, which was significantly higher than the figure of 5.89 µg/dL in Haojiang children after adjusting the covariates of age, height and weight (*P*<0.05). Regardless of sex, Children's BLLs in Guiyu were significantly higher than that in Haojiang (both *P*<0.05). Boys tended to have higher BLLs than girls, irrespectively of areas (both *P*<0.05). In addition, the subgroup analyses of BLLs exceeding 10 µg/dL showed the proportion (24.80%) of children with high BLLs in Guiyu was greater than the quantity (12.84%) in the reference group (*P*<0.05). The comparable findings in both genders were presented, irrespectively of areas (both *P*<0.05).

**Table 2:** Participant characteristics and blood lead levels (BLLs) of children in Guiyu and Haojiang.

	Guiyu	Haojiang	*P*-value
Age, mean±SD, year	4.82±1.21	4.38±0.95	<0.05
Sex, boys/girls, count	280/228	189/140	>0.05
Height, mean±SD, cm	105.95±8.39	104.52±8.07	<0.05
Weight, mean±SD, kg	17.25±3.17	17.72±3.38	<0.05
Head circumference, mean±SD, cm	50.25±2.91	50.11±1.61	>0.05
Chest circumference, mean±SD, cm	51.88±4.43	52.38±3.59	>0.05
BLLs, median (IQR), µg/dL			
Total	7.06 (4.71)	5.89 (3.54)	<0.05
Sex			
boys	7.23 (5.35)	6.32 (4.09)	<0.05
girls	6.83 (3.98)	5.58 (2.73)	<0.05
BLLs, counts (%),≥10 µg/dL			
Total	123 (24.80)	42 (12.84)	<0.05
Sex			
boys	80 (29.41)	29 (15.43)	<0.05
girls	43 (19.20)	13 (9.35)	<0.05

SD: standard deviation. IQR: interquartile range.

**Figure 2:**
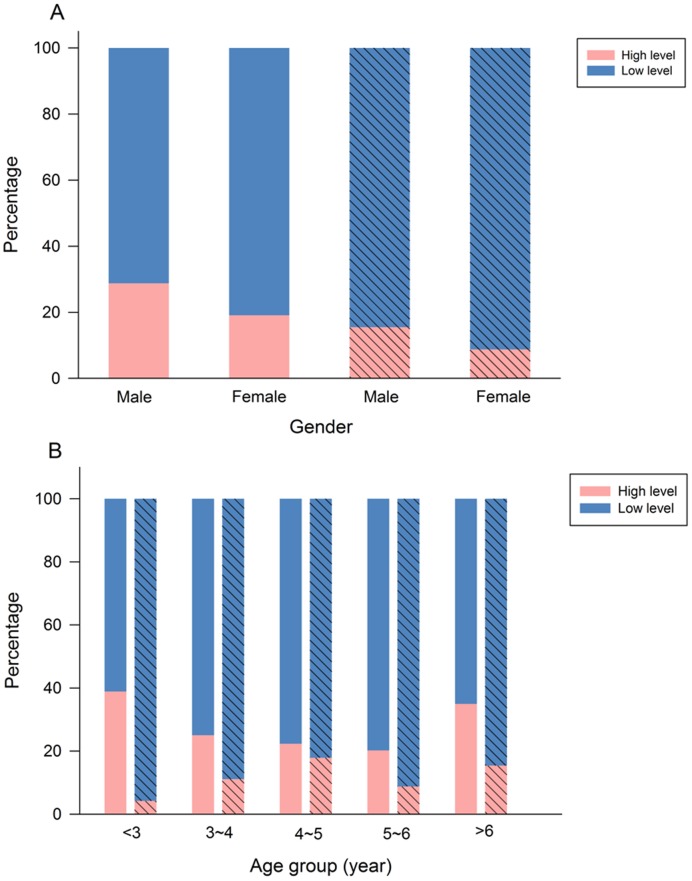
The percentage of children with high (≥10 µg/dL) and low (<10 µg/dL) blood lead levels in different sex (A) and age groups (B) in Guiyu and Haojiang. The bar with slash represents the Haojiang group.

Figure 2 presents the percentages of children with high and low BLLs in different sex and age groups in the two areas. Guiyu boys had the highest percentage of children with high BLLs exceeding 10 µg/dL, followed by Guiyu girls, Haojiang boys and Haojiang girls, respectively (Figure 2A). The percentages of children with high BLLs aged below 3 and above 6 years in Guiyu exceeded that in Haojiang (Figure 2B). The BLLs increasing with age were not presented in this study. Old children did not tend to have higher BLLs than the young. The distributions of children's age in Guiyu and Haojiang showed that most of children in the two areas were from 3–7 years and their distributions were similar (Figure S1 in File S1.).

### Risk factors associated with children's BLLs

**Figure 3:**
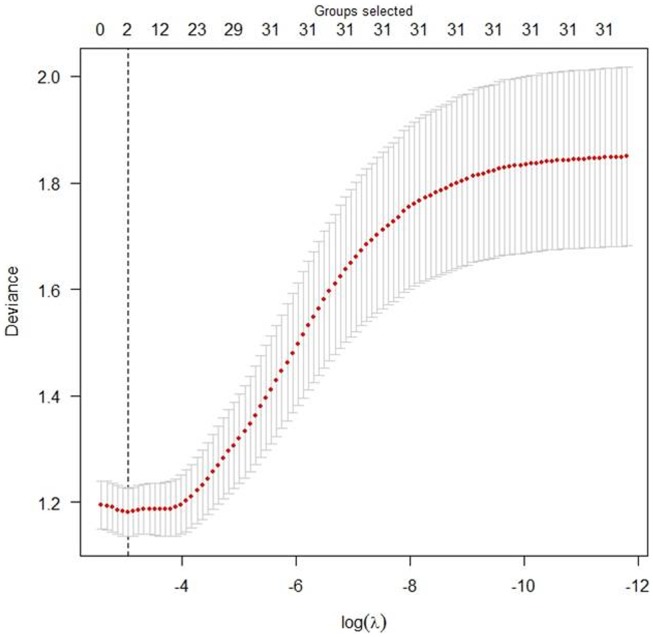
The deviance with error bar of the group Lasso logistic regression model using 10-fold cross-validation across different values of the tuning parameter 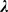 (log-scale). The total number of covariates is 31 and the corresponding number of dummy variables is 72. The optimal model is the one with deviance of 1.18 when 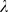 reaches 0.0471.

**Figure 4:**
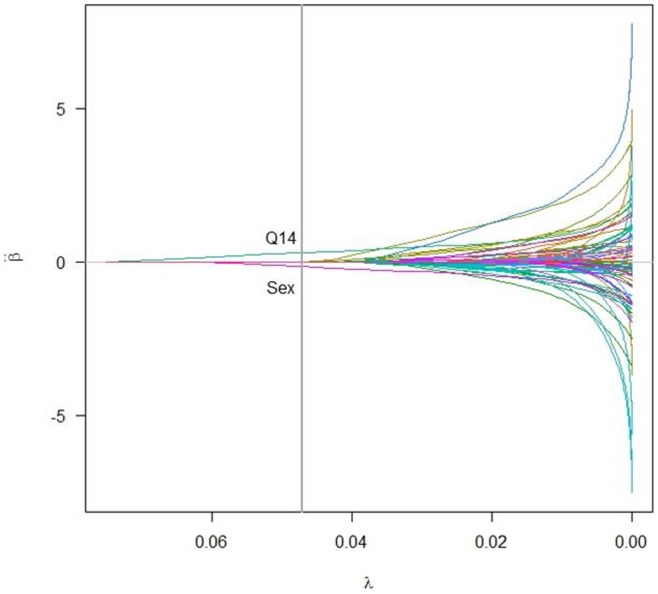
The path of the estimated coefficients over a grid of values for 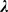, and a group of covariates including Sex and Q14 corresponding to 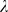 = 0.0471 was selected. Sex: The sex of child. Q14: Whether there are e-waste piles or recycling workshops within fifty meters around the house.

To identify risk factors associated with children's BLLs, the tuning parameter 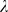 was initially determined in the group Lasso model. The 10-fold cross-validation was conducted to estimate the deviance for each model. Figure 3 shows the corresponding cross-validation deviances and the numbers of selected covariates at different values of 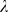 for the group Lasso models. As assessed, the optimal deviance was 1.18 with 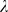 = 0.0471. Two groups of statistically significant covariates were selected when the tuning parameter reached 0.0471. The coefficient paths for the fitted group Lasso logistic models over a grid of values of 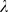 are presented in Figure 4. Two covariates including Sex and Q14 (Whether there are e-waste piles or recycling workshops within fifty meters around the house) corresponding to 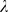 = 0.0471 were finally determined.

A general logistic regression model with all covariates was initially established as a comparison. The details of the selected covariates and their estimates for the logistic regression model are listed in Table 3. There were totally 14 factors (4 continuous covariates, 2 binary covariates and 8 ordinal covariates) selected by the logistic model. The estimates of some categories referenced on the first category were not statistically significant for some covariates. Indeed, the categorical covariates should be introduced as a whole factor in the model based on a group-wise selection. This result, from another perspective, may suggest the appropriateness of using the group Lasso for variable selection. Hence the group Lasso model was employed to select the most important covariates from all the candidates. According to the results of group Lasso, Sex [Lasso's 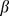 = −0.1612, OR (odds ratio) = 0.51] and Q14 [Lasso's 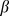 = 0.2874, OR = 2.28] were the protective and risk factors for children's BLLs in Guiyu, respectively. It was appropriate to find that the signs of the two estimates of Sex and Q14 from both the logistic model and the group Lasso logistic model were the same, reflecting the similar parameter effects on the response found by these two models. The estimate concludes that the risk of being high BLLs in Guiyu children increases when e-waste piles or recycling workshops occur within fifty meters around the house, and decreases in girls when compared with boys.

**Table 3:** The comparison of estimates of the logistic regression model with the significant selected factors and the group Lasso logistic regression model.

Variable name	Type	Logistic's 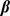	*P*-value	Lasso’s 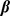	Odds Ratio (95% CI)
Age					
	1–3 years	-	-		
	3–5 years	−2.0486	0.0649		
	>5 years	−3.1721	0.0104		
Sex	Binary (boy and girl)	−1.3556	0.0004	−0.1612	0.5077 (0.3057, 0.8316)
Height	Continuous	0.1487	0.0045		
Weight	Continuous	−0.4500	0.0013		
Head circumference	Continuous	−0.1968	0.0038		
Chest circumference	Continuous	0.1470	0.0048		
The average time of the child eating bean products	None	-	-		
	1 to 3 times per month	1.4549	0.1638		
	1 to 3 times per week	1.8555	0.0864		
	At least 1 time per day	3.4762	0.0122		
The average time of the child eating canned foods	None	-	-		
	1 to 3 times per month	−0.1597	0.6743		
	1 to 3 times per week	2.6117	0.0143		
	At least 1 time per day	-	-		
The average time of the child taking oral solution with added calcium, iron or zinc	None	-	-		
	1 to 3 times per month	1.2097	0.0076		
	1 to 3 times per week	0.2516	0.7147		
	At least 1 time per day	−0.5410	0.5809		
The amount of cigarettes smoked by the family members every day	None	-	-		
	2 cigarettes per day	−1.5447	0.0085		
	10 cigarettes per day	−1.1433	0.0495		
	20 cigarettes per day	−2.52530	0.0002		
	More than 20 cigarettes per day	−3.1582	0.0002		
The distance between the house and the nearest road	Within 10 metres	-	-		
	Between 10 and 50 meters	1.1036	0.0440		
	Between 50 and 100 meters	−0.3032	0.5702		
	More than 100 meters	−0.6411	0.2700		
Whether there are e-waste recycling workshops within fifty meters around the house	Binary (none and yes)	1.8548	0.0001	0.2874	2.2810 (1.3740, 3.8658)
Mother's educational level	Illiteracy	-	-		
	Elementary education	-	-		
	Junior secondary education	−1.0233	0.0387		
	General secondary education	−1.7655	0.0128		
	Senior secondary education	0.9586	0.3446		
	Undergraduate education	−1.9343	0.0469		
The average time of the child contacting electronic wastes	None	-	-		
	Sometimes	0.7946	0.1052		
	Usually	−1.1587	0.3083		
	Always	1.3832	0.0173		

**Figure 5:**
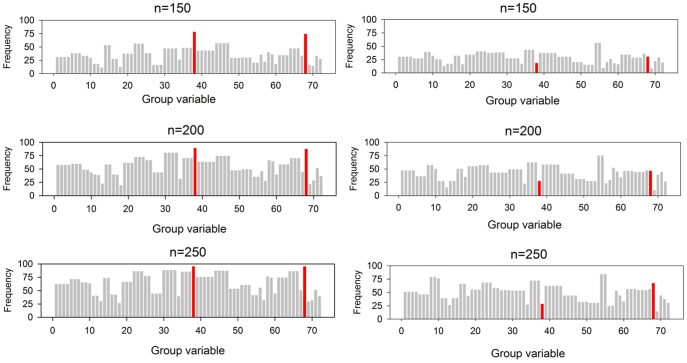
Group Lasso on the bootstrapped and permuted data. In each scenario, the frequency output of the bootstrapped data is left while the frequency output of the permuted data is right. The blue and red bars stand for the covariates of Sex and Q14, respectively. Sex: The sex of child. Q14: Whether there are e-waste piles or recycling workshops within fifty meters around the house.

We applied the group Lasso algorithm to the 100 sets of bootstrapped and permuted data with specified sample size (*N* = 150, 200 and 250) and record the frequency outputs of the covariates selected (Figure 5). We found that the real significant covariates of Sex and Q14 appeared most often in the bootstrapped data. It implied that these two factors were most robust in the group Lasso model in our study. Also, we permuted the binary response variable randomly in each data set to test whether the group Lasso model could select the correlation between the response and the covariates correctly. The frequency outputs in Figure 5 showed that the group Lasso selected covariates randomly on the permutated data, no matter how large the sample size was. After the permutation, the significant relations among the response and the predictors in the permutated data were removed. Therefore, the group Lasso method was confirmed to work well in our survey data.

## Discussion

This work provided an up-to-date assessment of children's BLLs from Guiyu and Haojiang in China during 2011–2013. Although the children have experienced an enjoyable decline in lead exposure over recent years, heavy metal poisoning is still a serious public health problem in Guiyu at present. The results of this study appear to support the role of e-waste recycling piles or workshops around the living area as a fundamental determinant in the elevated BLLs among Guiyu children. The lower BLLs among girls than boys, probably suggests a relatively less exposure to the environmental risk factors in the population. This analysis reinforces the importance of shifting e-waste recycling piles or workshops to non-populated areas as part of a comprehensive response to e-waste lead exposure control in Guiyu.

In Guiyu, e-waste recycling widely occurs in small scale family-based workshops, and the exposure to toxic lead from the activities of e-waste recycling has caused adverse effects on the health of local children and women. Accordingly, BLLs exceeding 10 µg/dL in children was defined as elevated BLLs that should take preventive measures [16]. The adverse effects of lead on children's health are well documented, but there is no safety margin at existing exposures for BLLs in children [22], [23]. Children are more sensitive to lead poisoning than adults [24], and it may due to an important truth that the developing central nervous system of young children is more vulnerable to toxicants than the mature one [25]. The elevated blood lead concentrations of children were associated with the decreased cognitive function [26], [27], the increased bone resorption and the delayed physical development [14], and intellectual impairment [28]. As a significant heavy metal toxicant in the river sediments in Guiyu, lead had notable impacts on the health of the residents and the local environment [29].

The monitoring of BLLs among Guiyu children has been performed by our laboratory since 2004, and the data showed that the estimated mean have declined from 15.3 µg/dL to 7.30 µg/dL over recent years [7], [11], [14], [15]. This present work found the median BLLs among Guiyu children during 2011–2013 was about 7.06 µg/dL, which was less than the estimates that previously reported. The BLLs of children in Guiyu appeared to decline, in part because local government made great efforts to control the pollution of e-waste recycling, and in part because we gave the lectures and guidance after sampling every year, and in part because the involved residents had an increasing awareness of self-protection. Health education about the means of preventing lead poisoning was provided through booklets, lectures, and posters by us to local parents and children during the surveys. In 2005, a large population-based study from China reported that the mean BLLs of 0–6 years old children from 15 selected cities was 5.95 µg/dL, and boys tended to have higher BLLs than girls [30]. The present BLLs data in this survey were notably higher than the reported national level. According to a recent report, from the end of the 1990s through 2009, China's average BLLs in urban children 0–6 years of age decreased from 7–10 µg/dL to 2.5–6 µg/dL [31]. Data from our studies indicated that the blood lead difference in Guiyu children is still higher than that in China overall. Although the percentage of children with high BLLs in Guiyu decreased, the public health impact of e-waste lead exposure is clear. As observed in this study, the BLLs of 7.06 µg/dL in Guiyu children was still significantly higher than that of 5.89 µg/dL in Haojiang children, suggesting that there was a close relationship between children's BLLs and the e-waste recycling activities. Children's lead poisoning prevention and controlling should be a long-term mission in Guiyu.

Among all the candidate factors investigated, the number of e-waste recycling piles or workshops near the house (OR = 2.28, 95% CI: [1.37, 3.87]) significantly contributed to the elevated BLLs for children in Guiyu. The lead concentration in dusts from e-waste recycling workshops was significantly higher in Guiyu than that in indoor dusts from other regions [14]. The large amount of e-waste recycling workshops around the living area might increase the risk of exposure to lead for children. In addition, girls tended to have less risk of high BLLs than boys (OR = 0.51, 95% CI: [0.31, 0.83]) in Guiyu. One could speculate that boys tended to more frequently have outdoor activities or engage in the activities of e-waste recycling, which might put the boys at high risk of exposure to lead. In regard to children's physical development, an inverse correlation between children's growth and high BLLs was characterized [32]. However, the relationship was not presented in the data for this survey. Further studies based on enlarged sample size should be carried out to determine the relationship between them. In addition, the high levels of lead load have significant adverse effects on behavioral neurological assessment scores of Guiyu neonates [33]. Information about the discrepancy in the scores between different genders in the region is not available currently. Regarding to Guiyu adult males, morbidity of genital diseases was high and the exposure to e-waste pollutants threatened their reproductive health [34].

To our knowledge, it is the first time to apply such a group-wise variable selection method to evaluate the risk factors associated with e-waste lead exposure for children. It might lead to new insights of using the grouped penalization method to select appropriate covariates in the research of environmental exposure assessment. In this study the number of questions in the questionnaire is 25, making the number of corresponding covariates up to 72 when dummy variables were used in the analysis. In order to identify the most important risk factors contributing to the elevated BLLs of children, a suitable variable selection methodology should be considered. In addition, it is inappropriate to build such a complex model with many covariates in prediction study because it may lead to the collinearity problem, which can severely distort the interpretation of a model and the role of each predictor [35]. The potential covariates should be determined after a quantitative variable selection. The Lasso method was proposed to identify risk factors of diseases because it outperformed in selecting the variables and estimating the corresponding parameters simultaneously [19]. Also, the Lasso method has been demonstrated to have a series of good asymptotic properties, such as the consistency [36], which strongly keeps meaningful variable selection.

However, the potential factors including demographic parameters and questionnaire items contain both continuous and categorical covariates in this study. Most of the covariates were measured in nominal or ordinal scales. It is reasonable to simultaneously select the dummy variables when they were generated by the same categorical covariates. All the dummy variables generated by the same factor should be determined at the same time to make them easily understandable. The group Lasso method, as an extension of Lasso, was introduced to deal with this problem by selecting whole factors at the group level instead of individual dummy variables [19], [37], [38]. Studies suggested the group Lasso method gave superior performance to the traditional stepwise backward elimination method in factor selection problems [39]. Owing to these strengths, the group Lasso logistic regression model was performed for our data. Furthermore, the analysis method presented in this work can be applied in the research of environmental exposure assessment.

In summary, the results of this study appear to support the role of e-waste recycling piles or workshops around the living area as a fundamental determinant in the elevated BLLs among Guiyu children. The lower BLLs among girls than boys, probably suggests a relatively less exposure to the environmental risk factors in the population. Children's lead poisoning prevention and controlling should be a long-term mission in Guiyu. Although some foundations supported by government programs have been channeled into the research of heavy metal risk assessment for the exposed population, broader recognition of e-waste lead exposure as a public health problem, a proactive management strategy taken to minimize e-waste production, effective protective measures from workshops and individuals are imperative if we want to keep the decline in children's BLLs ongoing in the future. More studies of lead exposure assessment as an essential way should be conducted to boost the health of local children in Guiyu.

Lead poisoning continues to be a major public health problem for local children in Guiyu. Coordinated and sustained efforts will be required to lessen the impact of e-waste lead exposure on Guiyu children now and in the future.

## Supporting Information

File S1 **This file contains Figure S1 and Table S1.** Figure S1. The age distributions of children from Guiyu and Haojiang. Table S1. The detailed description of the investigated factors from the survey.
(DOC)

## References

[1] Lundgren K (2012) The global impact of e-waste: addressing the challenge.

[2] UE P (2007) E-waste. Nairobi: UN Environment Programme.

[3] Noel-Brune M, Goldizen FC, Neira M, van den Berg M, Lewis N, et al (2013) Health effects of exposure to e-waste. The Lancet Global Health 1: e70. 25104155 10.1016/S2214-109X(13)70020-2

[4] Gavaghan H (2002) Lead, unsafe at any level. Bulletin of the World Health Organization 80: 82–82. 11884985 PMC2567638

[5] Tong S, Baghurst P, Sawyer M, Burns J, McMichael A (1998) Declining blood lead levels and changes in cognitive function during childhood. JAMA 280: 1915–1919. 9851476 10.1001/jama.280.22.1915

[6] Lanphear B, Hornung R, Khoury J, Yolton K, Baghurst P, et al (2005) Low-level environmental lead exposure and children's intellectual function:an international pooled analysis. Environ Health Perspect 113: 894. 16002379 10.1289/ehp.7688 PMC1257652

[7] Huo X, Peng L, Xu X, Zheng L, Qiu B, et al (2007) Elevated blood lead levels of children in Guiyu, an electronic waste recycling town in China. Environ Health Perspect 115: 1113–1117. 17637931 10.1289/ehp.9697 PMC1913570

[8] Wu KS, Xu XJ, Peng L, Liua JX, Guo YY, et al (2012) Association between maternal exposure to perfluorooctanoic acid (PFOA) from electronic waste recycling and neonatal health outcomes. Environment International 48: 1–8. 22820015 10.1016/j.envint.2012.06.018

[9] Xu X, Yang H, Chen A, Zhou Y, Wu K, et al (2012) Birth outcomes related to informal e-waste recycling in Guiyu, China. Reprod Toxicol 33: 94–98. 22198181 10.1016/j.reprotox.2011.12.006

[10] Zheng GN, Xu XJ, Li B, Wu KS, Yekeen TA, et al (2013) Association between lung function in school children and exposure to three transition metals from an e-waste recycling area. Journal of Exposure Science and Environmental Epidemiology 23: 67–72. 22854517 10.1038/jes.2012.84

[11] Liu JX, Xu XJ, Wu KS, Piao ZX, Huang JR, et al (2011) Association between lead exposure from electronic waste recycling and child temperament alterations. Neurotoxicology 32: 458–464. 21477618 10.1016/j.neuro.2011.03.012

[12] Peng ML, Hao JH, Su PY, Ding JL, Xu XR (2011) Children's blood lead levels in seven districts in Hefei Province from 2008 to 2009 and its related factors [in Chinese]. Zhong Guo Er Tong Bao Jian Za Zhi 19: 740–742.

[13] Qi QP, Yang YW, Yao XY, Ding L, Wang W, et al (2002) The survey of blood lead levels in Chinese urban children [in Chinese]. Zhong Hua Liu Xing Bing Xue Za Zhi 23: 162–166. 12411080

[14] Zheng LK, Wu KS, Li Y, Qi ZL, Han D, et al (2008) Blood lead and cadmium levels and relevant factors among children from an e-waste recycling town in China. Environmental Research 108: 15–20. 18514186 10.1016/j.envres.2008.04.002

[15] Yang H, Huo X, Yekeen TA, Zheng QJ, Zheng MH, et al (2013) Effects of lead and cadmium exposure from electronic waste on child physical growth. Environmental Science and Pollution Research 20: 4441–4447. 23247522 10.1007/s11356-012-1366-2

[16] Binns HJ, Campbell C, Brown MJ (2007) Interpreting and managing blood lead levels of less than 10 mu g/dL in children and reducing childhood exposure to lead: Recommendations of the Centers for Disease Control and Prevention advisory committee on childhood lead poisoning prevention. Pediatrics 120: E1285–E1298. 17974722 10.1542/peds.2005-1770

[17] Tibshirani R (2011) Regression shrinkage and selection via the lasso: a retrospective. Journal of the Royal Statistical Society Series B-Statistical Methodology 73: 273–282.

[18] Li Y, Qin YC, Xie YM, Tian F (2013) Grouped penalization estimation of the osteoporosis data in the traditional Chinese medicine. Journal of Applied Statistics 40: 699–711.

[19] Meier L, van de Geer SA, Buhlmann P (2008) The group lasso for logistic regression. Journal of the Royal Statistical Society Series B-Statistical Methodology 70: 53–71.

[20] Huang J, Breheny P, Ma SG (2012) A Selective Review of Group Selection in High-Dimensional Models. Statistical Science 27: 481–499. 10.1214/12-STS392 PMC3810358 24174707

[21] Team RDC (2005) R: A language and environment for statistical computing. ISBN 3-900051-07-0. R Foundation for Statistical Computing. Vienna, Austria, 2013. Available: http://www.R-project.org.

[22] Grigg J (2004) Environmental toxins; their impact on children's health. Archives of disease in childhood 89: 244–250. 14977703 10.1136/adc.2002.022202 PMC1719840

[23] Koller K, Brown T, Spurgeon A, Levy L (2004) Recent developments in low-level lead exposure and intellectual impairment in children. Environ Health Perspect 112: 987. 15198918 10.1289/ehp.6941 PMC1247191

[24] Jain NB, Hu H (2006) Childhood correlates of blood lead levels in Mumbai and Delhi. Environ Health Perspect 114: 466–470. 16507473 10.1289/ehp.8399 PMC1392244

[25] Needleman H (2004) Lead poisoning. Annu Rev Med 55: 209–222. 14746518 10.1146/annurev.med.55.091902.103653

[26] Bellinger D, Leviton A, Waternaux C, Needleman H, Rabinowitz M (1987) Longitudinal analyses of prenatal and postnatal lead exposure and early cognitive development. N Engl J Med 316: 1037–1043. 3561456 10.1056/NEJM198704233161701

[27] McMichael AJ, Baghurst PA, Wigg NR, Vimpani GV, Robertson EF, et al (1998) Port Pirie Cohort Study: environmental exposure to lead and children's abilities at the age of four years. N Engl J Med 319: 468–75. 10.1056/NEJM198808253190803 3405253

[28] Canfield RL, Henderson Jr CR, Cory-Slechta DA, Cox C, Jusko TA, et al (2003) Intellectual impairment in children with blood lead concentrations below 10 µg per deciliter. N Engl J Med 348: 1517–1526. 12700371 10.1056/NEJMoa022848 PMC4046839

[29] Wong CSC, Wu SC, Duzgoren-Aydin NS, Aydin A, Wong MH (2007) Trace metal contamination of sediments in an e-waste processing village in China. Environmental Pollution 145: 434–442. 16824655 10.1016/j.envpol.2006.05.017

[30] Zhang S, Dai Y, Xie X, Fan Z, Tan Z (2005) Study on blood lead level and related risk factors among children aged 0–6 years in 15 cities [in China]. Zhonghua liuxingbingxue zazhi 26: 651. 16471210

[31] Yan CH, Xu J, Shen XM (2013) Childhood Lead Poisoning in China: Challenges and Opportunities. Environ Health Perspect 121: A294. 24218672 10.1289/ehp.1307558 PMC3801475

[32] Schwartz J, Angle C, Pitcher H (1986) Relationship between childhood blood lead levels and stature. Pediatrics 77: 281–288. 3951909

[33] Li Y, Xu X, Wu K, Chen G, Liu J, et al (2008) Monitoring of lead load and its effect on neonatal behavioral neurological assessment scores in Guiyu, an electronic waste recycling town in China. J Environ Monit 10: 1233–8. 19244648 10.1039/b804959a

[34] Xu X, Zhang Y, Yekeen TA, Li Y, Zhuang B, et al (2014) Increase male genital diseases morbidity linked to informal electronic waste recycling in Guiyu, China. Environ Sci Pollut Res Int 21: 3540–5. 24271725 10.1007/s11356-013-2289-2

[35] Woolston A, Tu YK, Baxter PD, Gilthorpe MS (2010) A New Index to Assess the Impact of Collinearity in Epidemiological Research. Journal of Epidemiology and Community Health 64: A59–A59.

[36] Knight K, Fu W (2000) Asymptotics for lasso-type estimators. Annals of Statistics: 1356–1378.

[37] Duarte MF, Bajwa WU, Calderbank R (2011) The performance of group Lasso for linear regression of grouped variables. Duke University, Dept Computer Science, Durham, NC, Technical Report TR-2010-10.

[38] Zhou H, Alexander DH, Sehl ME, Sinsheimer JS, Lange K (2011) Penalized regression for genome-wide association screening of sequence data. World Scientific: pp. 106–117. 10.1142/9789814335058_0012 PMC5049883 21121038

[39] Yuan M, Lin Y (2006) Model selection and estimation in regression with grouped variables. Journal of the Royal Statistical Society Series B-Statistical Methodology 68: 49–67.

