# Supplementary material for: Blood Lead Levels and Associated Factors among Children in Guiyu of China: A Population-Based Study
Source: PLoS One. 2014 Aug 19;9(8):e105470. doi: 10.1371/journal.pone.0105470 (PMC4138148; doi:10.1371/journal.pone.0105470)
Supplement: File S1 — This file contains Figure S1 and Table S1. Figure S1. The age distributions of children from Guiyu and Haojiang. Table S1. The detailed description of the investigated factors from the survey. (DOC) [file pone.0105470.s001.doc]

**Supporting Information File S1.**

**Figure S1** The age distributions of children from Guiyu and Haojiang.


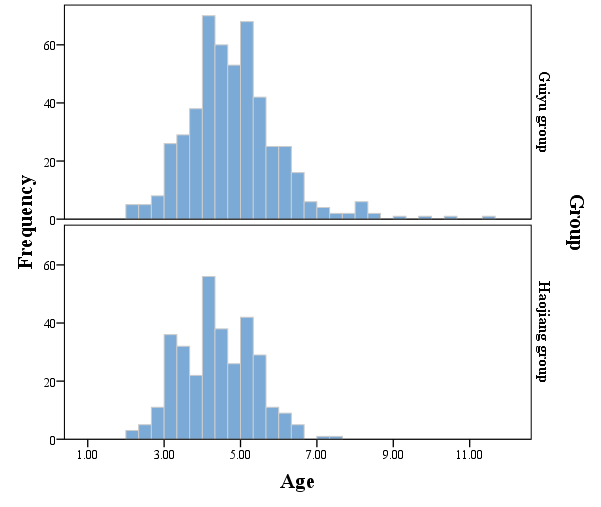


**Table S1** The detailed description of the investigated factors from the survey.

| Variable | Type | Details |
| --- | --- | --- |
| Sex | Binary | The sex of child |
| Age | Continuous | The age of child |
| Height | Continuous | The height of child |
| Weight | Continuous | The weight of child |
| HM | Continuous | The head circumference of child |
| CM | Continuous | The chest circumference of child |
| Q1 | Categorical | The average time of the child playing outside every day |
| Q2 | Categorical | Whether the child often washes hands before eating meals or snacks |
| Q3 | Categorical | Whether the child often bites or sucks fingernails |
| Q4 | Categorical | Whether the child often sucks or bites pencils or erasers |
| Q5 | Categorical | Whether the child often sucks or bites toys |
| Q6 | Categorical | The average time of the child eating preserved eggs every year |
| Q7 | Categorical | The average time of the child eating dairy products every year |
| Q8 | Categorical | The average time of the child eating bean products every year |
| Q9 | Categorical | The average time of the child eating canned foods every year |
| Q10 | Categorical | The average time of the child taking oral solution with added calcium, iron or zinc every year |
| Q11 | Categorical | Whether the family members smoke. If they somke, the amount of cigarettes consumed every day |
| Q12 | Binary | Whether the house is a workplace |
| Q13 | Categorical | The distance between the house and the nearest road |
| Q14 | Binary | Whether there are e-waste piles or recycling workshops within fifty meters around the house |
| Q15 | Categorical | Father's educational level |
| Q16 | Categorical | Mother's educational level |
| Q17 | Categorical | The time of father living in the area |
| Q18 | Categorical | The time of mother lives in the area |
| Q19 | Categorical | The time of the child living in the area |
| Q20 | Binary | Whether father often takes off work clothes before touching the child |
| Q21 | Binary | Whether mother often takes off work clothes before touching the child |
| Q22 | Categorical | The main source of drinking water of the house |
| Q23 | Binary | Whether the child often partial eclipse |
| Q24 | Categorical | The average time of the child taking pills with added Vitamin C, D and E |
| Q25 | Categorical | The average time of the child contacting electronic wastes |
